# Free glutaraldehyde gelatin microsphere loaded mesenchymal stem cells alleviate osteoarthritis by promoting *Ext1* expression

**DOI:** 10.7150/thno.109468

**Published:** 2025-03-10

**Authors:** Ye Yuan, Longsheng Xu, Yu Zhao, Tizhen Yan, Baobiao Zhuo, Fan Bu, Qian Cai, Youjia Xu, Qing Bi, Yu Tong, Dan Xing, Di Sun, Jianhao Lin

**Affiliations:** 1 Arthritis Clinic & Research Center, Peking University People's Hospital, Peking University, Beijing, 100044, China; Arthritis Institute, Peking University, Beijing 100044, China.; 2 Department of Anesthesiology and Pain Research Center, The Affiliated Hospital of Jiaxing University, Jiaxing 314001, Zhejiang, China.; 3 Department of Orthopaedics, Shanxi Key Laboratory of Bone and Soft Tissue Injury Repair, the Second Hospital of Shanxi Medical University, Taiyuan 030001, Shanxi, China.; 4 Institute of Reproduction and Genetics, Dongguan Maternal and Child Health Care Hospital; Pediatric Orthopedics, Dongguan Maternal and Child Health Care Hospital, Dongguan 523120, Guangdong, China.; 5 Department of Neurology & Psychology, Shenzhen Traditional Chinese Medicine Hospital, the Fourth Clinical Medical College of Guangzhou University of Chinese Medicine, Shenzhen 518033, Guangdong, China.; 6 State Key Lab of Structural Chemistry, Fujian Institute of Research on the Structure of Matter, Chinese Academy of Sciences, Fuzhou 350002, Fujian, China; 7 Department of Orthopaedics, Second Affiliated Hospital of Soochow University, Osteoporosis Research Institute of Soochow University, No. 1055 Sanxiang Road, Suzhou 215000, Jiangsu, China.; 8 Center for Rehabilitation Medicine, Department of Orthopedics, Zhejiang Provincial People's Hospital (Affiliated People's Hospital, Hangzhou Medical College), Hangzhou 310014, Zhejiang, China.; 9 Key Laboratory of Photochemical Conversion and Optoelectronic Material, Technical Institute of Physics and Chemistry, Chinese Academy of Sciences, Beijing 100190, China.

**Keywords:** Osteoarthritis, Gelatin microsphere, Mesenchymal stem cells, *Ext1*, Immune response

## Abstract

**Rationale:** Osteoarthritis (OA) is a chronic joint disorder with limited treatment efficacy, necessitating innovative therapeutic strategies. This study explores one-pot-synthesized gelatin microspheres devoid of glutaraldehyde as a novel biomaterial for OA management. Focusing on the *Ext1* gene, critical for cartilage development and downregulated in OA, we investigated its restoration and immune regulation using gelatin microspheres cultured with mesenchymal stem cells (MSCs).

**Methods:** OA patients undergoing knee replacement surgery have their lateral compartment (remote zone) and medial compartment (lesion zone) cartilage collected for transcriptomic testing. The differential gene *Ext1* is identified, and the expression of immune regulatory genes is examined. MSCs were cultured with gelatin microspheres to evaluate their compatibility and ability to promote cell attachment. The effects of the gelatin microspheres on *Ext1* gene overexpression, immune regulation, and OA symptom mitigation were investigated through *in vitro* and *in vivo* experiments.

**Results:** OA patients exhibit decreased expression of the *Ext1* gene in the medial compartment (lesion zone) cartilage area, accompanied by abnormal expression of immune regulatory genes. The study demonstrated that the gelatin microspheres exhibited excellent compatibility with MSCs and facilitated their attachment. Culturing MSCs with the microspheres led to enhanced overexpression of the *Ext1* gene, which is crucial for cartilage growth and development. Additionally, the microspheres regulated immune responses, contributing to a reduction in OA symptoms.

**Conclusion:** This study introduces an innovative therapeutic strategy for osteoarthritis using gelatin microspheres cultured with MSCs. By promoting *Ext1* gene overexpression and regulating immune responses, these microspheres effectively mitigate OA symptoms. The findings highlight the potential of this biomaterial as a promising treatment option for OA.

## Introduction

Osteoarthritis (OA) is a common chronic joint disease that predominantly affects middle-aged and older individuals, severely impacting the quality of life of patients. It is estimated that more than 240 million people worldwide are affected by OA, with an incidence rate of approximately 15% in China. With the aging population, the incidence rate is on the rise. Currently, conservative treatments mainly include medication and physical rehabilitation. For patients with severe osteoarthritis, surgery is an effective method. However, conservative treatments can only alleviate symptoms, while surgical treatments are invasive and procedures such as joint replacement have a limited lifespan^1^. Therefore, it is necessary to delve into the pathogenesis of OA and develop novel treatment methods.

Currently, there is no effective treatment for OA except for late stage total joint replacement surgery. MSCs are a type of adult stem cell with multiple differentiation abilities and immune regulatory potential^2^. Under specific conditions, they can promote their differentiation into chondrocytes, maintaining their function and stability. MSCs can effectively regulate the cartilage micro-environment, promote cartilage regeneration, and alleviate symptoms of osteoarthritis. They are a promising cell source for OA treatment. Animal and clinical studies have shown that whether used alone or in combination with biomaterials, extracellular vesicles can be secreted to treat OA in various ways. Extracellular vesicles can carry bioactive factors such as proteins, lipids, mRNA, miRNA, etc^3^. Previous studies have shown that using MSCs to treat OA may be effective, but the therapeutic effect and mechanism of action are unclear^4^. OA is related to immune regulation^5^, and these characteristics offer new strategies for the treatment of osteoarthritis^6^, ^7^.

*Ext1* gene, crucial for cartilage growth and heparan sulfate synthesis, is linked to hereditary multiple exostoses^8^, ^9^. Its mutations may disrupt growth factor signaling and cartilage maintenance, potentially influencing osteoarthritis development via the Wnt/β-catenin pathway^10^, ^11^. The exact role in OA pathogenesis requires further study^12^.

It is crucial to effectively carry this gene and choose gelatin microsphere as a carrier that promotes bone growth and adherent cells. Gelatin have shown great potential in the treatment of OA because of anti-inflammatory, non-toxicity, low cost, low immunogenicity, good biodegradability and biocompatibility^13^. Gelatin microsphere is a micro nano scale spherical particle, used in drugs. Gelatin microsphere is prepared by spray drying, electrospray, microfluidics fabrication and emulsification^14^.

**Figure 1:**
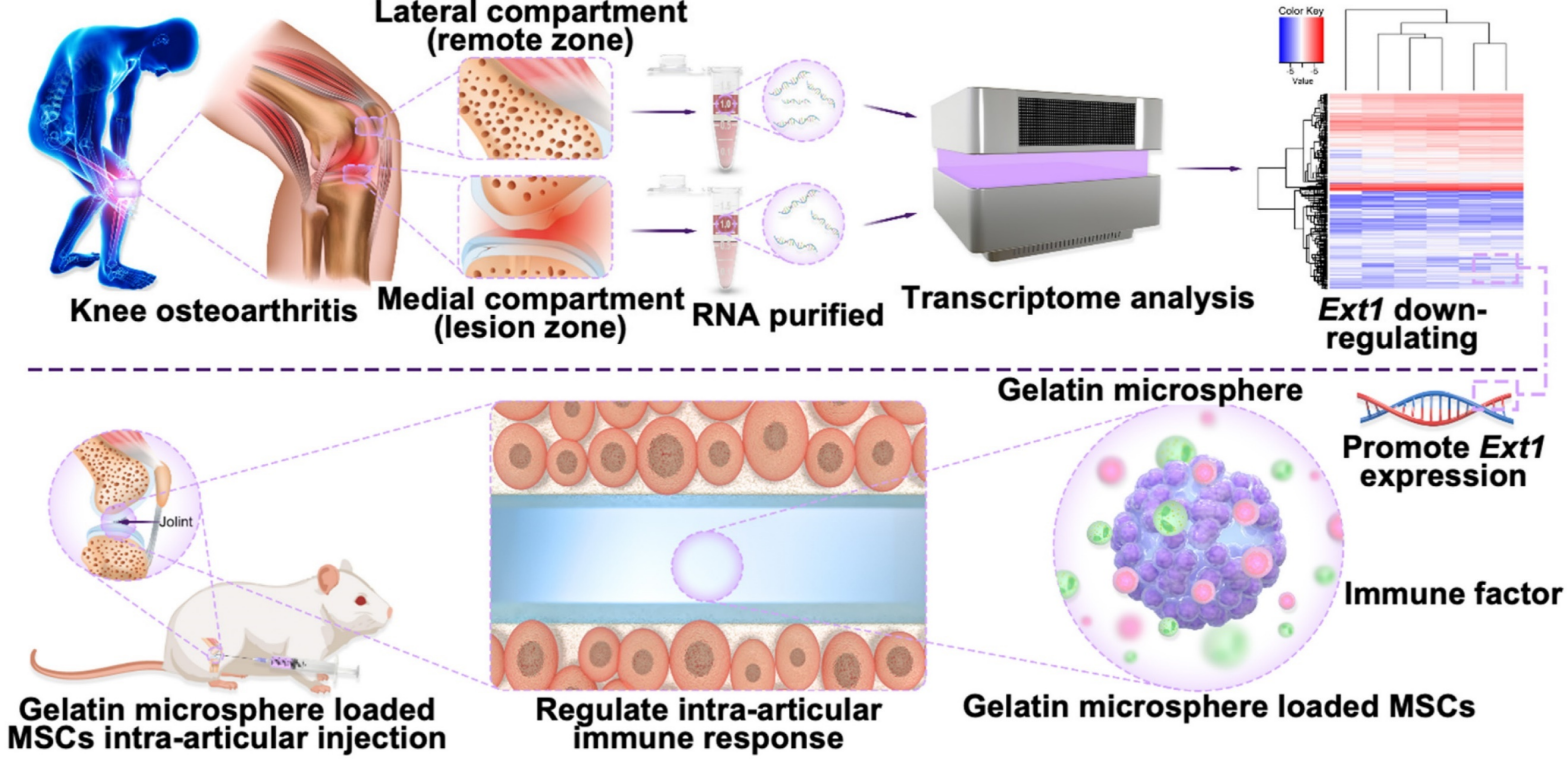
Gelatin microsphere shows promise in treating osteoarthritis by enhancing Ext1 gene expression and modulating immune responses through mesenchymal stem cell adhesion. This novel approach offers an innovative therapeutic strategy for OA management.

In this study, downregulation of *Ext1* gene was found in the cartilage weight-bearing area of OA patients in human samples; Furthermore, a three-dimensional system of gelatin microsphere carrying MSCs was constructed to promote *Ext1* expression and regulate immune response for the treatment of OA (Figure 1). Gelatin microsphere has two main advantages: (1) The porous structure of gelatin microsphere promotes cell adhesion and the transfer of nutrients and metabolic waste and simulating the three-dimensional real environment inside the body, it can be developed into organoids in the future; (2) The porous structure of gelatin microsphere effectively load stem cells to overexpress *Ext1* gene, regulate immune response, and alleviate OA. Following the downregulation of the *Ext1* gene in the cartilage weight-bearing area of OA patients in human sample; Furthermore, a three-dimensional system of gelatin microsphere carrying MSC cells was constructed to promote *Ext1* expression and regulate immune response for the treatment of OA, which providing a new target for future clinical treatment of OA.

## Materials

Gelatin were purchased from sigmal, USA. Span 80 and acetone were purchased from Aladdin, China. DiR Iodide deep red cell membrane fluorescent probe was purchased from Yeasen, China. DAPI staining solution was purchased from Beyotime, China. iFluor 488 phalloidin was purchased from Yeasen, China. Calcein AM/PI was purchased from from Beyotime, China. Pholloidin was purchased from Cytoskeleton, US. Mouse IFN-gamma recombinant protein was purchased from Thermo Fisher Scientific, US. Type II collagenase from Gibco, US. α-MEM from Gibco, US. PFA and Triton X-100 were from Beyotime Biotechnology, China.

## Methods

### Human osteoarthritis sample collection

This study was approved by the Ethics Committee of Peking University People's Hospital (2019 phb098-01). Discarded cartilage tissue was extracted from patients undergoing total knee arthroplasty, with the following inclusion criteria: age 68±6.48 years (mean±SD), and clinical diagnosis based on radiological Kellgren-Lawrence grade 3. Exclusion criteria included infectious arthritis or rheumatoid arthritis. According to the International Cartilage Repair Society (ICRS) criteria, the remote area was defined as the central region of the tibial plateau with an ICRS grade I, which is a non-weight-bearing area. The lesion area was located in the lateral tibial plateau with ICRS grades II and III, which are weight-bearing areas.

### Transcriptomic analysis

After obtaining human cartilage samples, the chondrocytes are digested as follows: Cartilage samples are taken from both the remote and lesion areas. Each clinical specimen is subjected to consecutive enzymatic digestion with 0.2% type II collagenase at 37°C for 10 h, washed in phosphate-buffered saline (PBS), filtered through a 100mm nylon mesh, and RNA is collected using Trizol reagent for transcriptomic analysis. RNA is collected using Trizol reagent for transcriptomic analysis.

Data analysis and plotting were all conducted in the R language environment. Differential gene screening was performed by the R package limma, with the screening criteria being log_2_ fold change values greater than 1 or less than -1, and p-values less than 0.05. The volcano plot for differential genes was created using the R package ggplot2, and the heatmap for differential genes was completed by the R package heatmap. Enrichment analysis was carried out by the R package cluster Profiler, and the GO and KEGG enrichment result plots were created using the R package ggplot2.

### Gelatin microsphere

1.5g of gelatin powder dissolved in 10mL of deionized water and stirred continuously at 60°C to prepare a 15% (w/v) gelatin solution as the aqueous phase. Span 80 (0.2mL) was heated to 60 °C in 20mL of paraffin oil to form an oil phase. The gelatin solution is slowly dropped into the oil phase solution for homogenization to form emulsion droplets in the W/O lotion. In the process of cooling to 4°C, the emulsion drops become gelatin microsphere, washed with acetone three times to remove the excess oil phase substances, washed with deionized water three times, and then dried to obtain the gelatin microsphere before modification. Gelatin is irradiated with ultraviolet light for 2 h before use in cell culture to ensure sterility.

### Scanning Electron Microscopy (SEM)

The gelatin microsphere is subjected to freeze-drying and surface-treated and sputter-coated with gold. The prepared sample is placed on the SEM sample stage, ensuring stability. In the SEM, appropriate parameters such as acceleration voltage, working distance, and probe current are set. The sample is then scanned, and secondary electron or backscattered electron images are collected. The collected images are analyzed using the image analysis software equipped with the SEM to study the microstructure and morphology of the gelatin microsphere.

### MSCs culture with gelatin microsphere

MSCs, isolated from human embryonic stem cells, were obtained from CytoNiche Biotech. MSCs were grown in α-MEM and contained 10% FBS. Gelatin microsphere were sterilized under ultraviolet radiation for 2 h before cell seeding. MSCs were washed, counted, centrifuged, and resuspended. Cells were seeded into gelatin microsphere at a final concentration of 5×10^5^ cells per scaffold in 48-well plates, and were allowed to attach for 2 h at 37°C. Finally, 500mL of growth medium was added to each well and seeded scaffolds were cultured at 37°C with 5% CO_2_ for up to 9 days, with medium changed every 3 days.

### Cell Immunofluorescence

Collect cell samples and fix them with 4% PFA, then wash with PBS (phosphate-buffered saline). Clean with Triton X-100, and block antibody binding sites to cell surfaces with non-specific interactions using bovine serum albumin (BSA). Add specific antibodies and incubate for a period of time at the appropriate temperature. Wash the cells with PBS to remove unbound antibodies. Stain the cell nuclei with DAPI (4',6-diamidino-2-phenylindole) dye. Observe the samples with a confocal fluorescence microscope and use imaging software for image acquisition and analysis.

### Cell culture with IFN-γ treatment

The aforementioned source of MSCs was passaged and transferred into a 6-well plate. MSCs were seeded at a density of 3×10^5^ cells/well. The control group used traditional two-dimensional culture for MSCs (2D culture), while the experimental group employed three-dimensional culture with gelatin microspheres for MSCs (3D culture). Both groups were induced with IFN-γ to detect the regulation of immune factors.

After adherence for 24 h, IFN-γ 50ng/mL (Beyotime Biotechnology) was added into the culture medium to culture for 24 h. Cells and conditioned medium were harvested after IFN-γ stimulation for Luminex liquid suspension chip immune cytokine analysis.

### Animal osteoarthritis model

This study was approved by the Ethics Committee of Peking University People's Hospital (RDJP2023-21). SPF-grade SD rats were randomly divided into three groups: the control group (Ctrl), the osteoarthritis group (OA), and the osteoarthritis treatment group (OA+GM), with 5 rats in each group. Eight-week-old SD rats were anesthetized, and the surgical area of the knee joint was shaved and sterilized. An appropriate skin incision was made at the knee joint to expose the joint. Through blunt dissection, the anterior structures of the knee joint, including the patella, patellar tendon, and joint capsule, were exposed. The anterior cruciate ligament (ACL) was transected, and the complete severing of the ligament and the reduced joint stability were confirmed by moving the knee joint. The joint capsule and skin incision were sutured with absorbable or non-absorbable sutures. Appropriate pain management and antibiotic prophylaxis were provided to the rats after surgery. The rats needed to move around independently after recovering from anesthesia. Cell therapy was injected at different time points postoperatively, and data related to joint stability, cartilage degeneration, and inflammatory response were collected and analyzed.

### Histology examination

Tissues were fixed in 10% formalin overnight, decalcified by 20% EDTA for 2 weeks, and processed for paraffin sectioning. Tibiae were collected and fixed overnight at 4 °C and embedded undecalcified in methyl methacrylate. Sections 5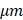 thick were processed for staining. Details were shown in our previous work^15^.

### Statistical analysis

Experimental data were presented as means±standard deviations (SDs), which was evaluated using Student's t tests, while one-way ANOVA was employed for multiple group analyses, followed by post-hoc tests for detailed pairwise comparisons. These analyses were conducted using GraphPad Prism software (version 9.5; La Jolla, California, USA). For ANOVA, post-hoc comparisons were performed using Tukey's multiple comparisons test, as appropriate, to adjust for multiple testing. A p-value of less than 0.05 was deemed to indicate statistical significance.

## Results and Discussion

### Downregulated expression of the cartilage *Ext1* gene in patients with osteoarthritis

**Figure 2:**
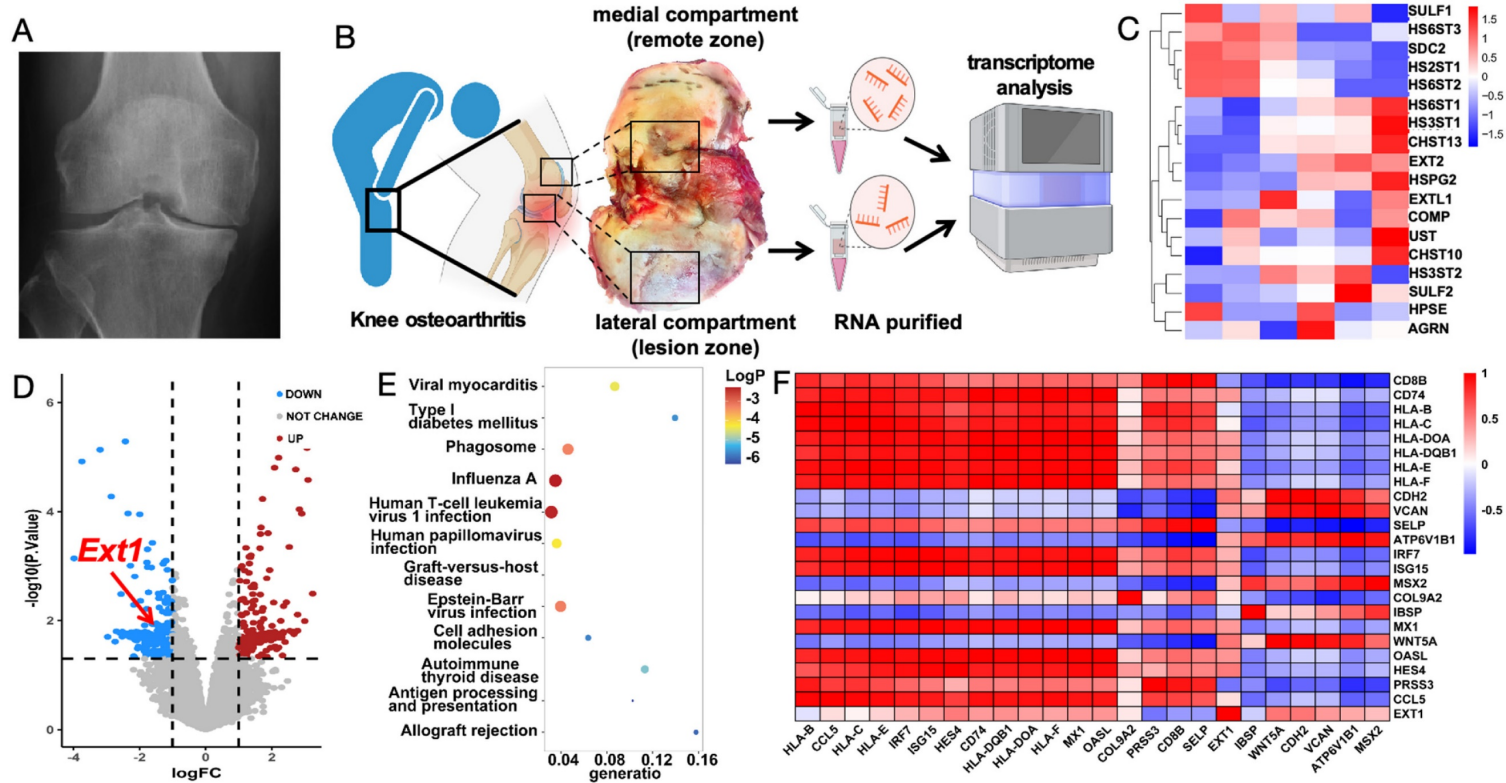
Osteoarthritis patients exhibit abnormal expression of the Ext1 gene and immune response. (A) Representative X-ray image of a knee with osteoarthritis in a patient. (B) Cartilage tissue samples were procured from the medial (Control group) and lateral (cartilage degeneration) compartments of the knee during surgical procedures in patients with knee osteoarthritis. RNA was subsequently extracted from these samples for transcriptomic analysis. (C) Heatmap illustrating the differential expression patterns of the transcriptome in knee joint cartilage tissue. (D) Volcano plot depicting the significant changes in gene expression levels in the knee joint cartilage tissue transcriptome. (E) Table summarizing the KEGG pathways enriched in the transcriptome of knee joint cartilage tissue, with associated gene counts and significance levels. (F) Further selection of immune regulatory genes previously studied and believed to be associated with the pathogenesis of osteoarthritis was conducted to create a correlation heatmap.

To explore the pathogenesis of osteoarthritis, cartilage samples from the weight-bearing and non-weight-bearing regions of the distal femoral condyles were first collected during surgery on patients with late-stage knee osteoarthritis (Figure 2A). Arthritis cartilage damage often occurs in weight-bearing areas, where severe cartilage surface wear and cartilage degeneration can be observed. Chondrocytes from both the non-weight-bearing and weight-bearing areas were digested, and cellular RNA was extracted for transcriptomic analysis. KEGG analysis revealed that the downregulation of genes such as *Ext1* is associated with the regulation of immune response (Figure 2B-C). Compared to the non-weight-bearing area, the expression of *Ext1* and genes related to the regulation of extracellular matrix glycan synthesis were downregulated in chondrocytes from the weight-bearing area (Figure 2D). RT-PCR varified transcriptomic results (Figure S1). Functional enrichment analysis suggests that, in addition to pathways related to the *Ext1* gene, changes have occurred in the expression of genes associated with immune regulation (Figure 2E). Further selection of immune regulatory genes previously studied and believed to be associated with the pathogenesis of osteoarthritis was conducted to create a correlation heatmap (Figure 2F). The above findings indicate that the degeneration of osteoarthritis cartilage damage is related to the disruption of immune response regulation caused by the downregulated expression of *Ext1* gene.

Previous studies have found that *Ext1* is involved in the growth and development of chondrocytes. Hereditary multiple osteochondromas is an autosomal dominant genetic disease characterized by multiple cartilaginous overgrowths at the ends of long bones and flat bones. *Ext1* gene mutations have been found in patients with hereditary multiple osteochondromas^16^. Conditional knockout *Ext1* gene (*Ext1^flox/flox^*; Col2CreERT) mice were injected with tamoxifen on the 5th day after birth to ablate *Ext1* in cartilage, and it was found that *Ext1* is continuously needed for the postnatal growth and tissue of long bones and their adjacent joints. *Ext1* deficiency can cause defects in skeletal diseases, including trabecular bone loss, osteoarthritis, and hereditary multiple exostoses^17^. Heparan sulfate proteins are encoded by *Ext1* gene, and heparan sulfate is an important component of cell surface and matrix proteoglycans, including syndecans and perlecan. Heparan sulfate chains can specifically interact with signaling proteins, including bone morphogenetic proteins (BMPs), through their heparan sulfate binding domains^18^. Heparan sulfate deficiencies in hereditary multiple exostoses may lead to widespread local BMP signaling and alterations in BMP receptor dynamics, triggering excessive cellular responses and exostosis formation^19^. Heparan sulfate regulated by *Ext1* gene plays a key role in various biological functions, including skeletal development^20^.

Some studies have also found the relationship between heparan sulfate proteins and osteoarthritis. Using Prg4-Cre transgenic mice to generate conditional knockout of *Ext1* in cartilage tissue (*Ext1*-cko mice), the lack of heparan sulfate in articular chondrocytes leads to chondrocyte hypertrophy, which is partially contributed by the upregulation of BMP/Smad signaling. Heparan sulfate may play an important role in maintaining the cartilage matrix by regulating BMP signaling^21^. Heparanase is the only mammalian enzyme with endoglycosidase activity for degrading heparan sulfate. It is active in osteoarthritis cartilage and induces a catabolic response in human primary chondrocytes, which is at least partially due to the release of soluble growth factors such as FGF2^22^. Increased sulfation of heparan sulfate chains in the cartilage of osteoarthritis patients alters the binding characteristics of heparin-binding proteins and their biological effects on the chondrocyte phenotype. Therefore, the modified heparan sulfate present in the altered cartilage may be a new therapeutic target for OA^23^.

### MSCs were cultured with three-dimensional gelatin microsphere

**Figure 3:**
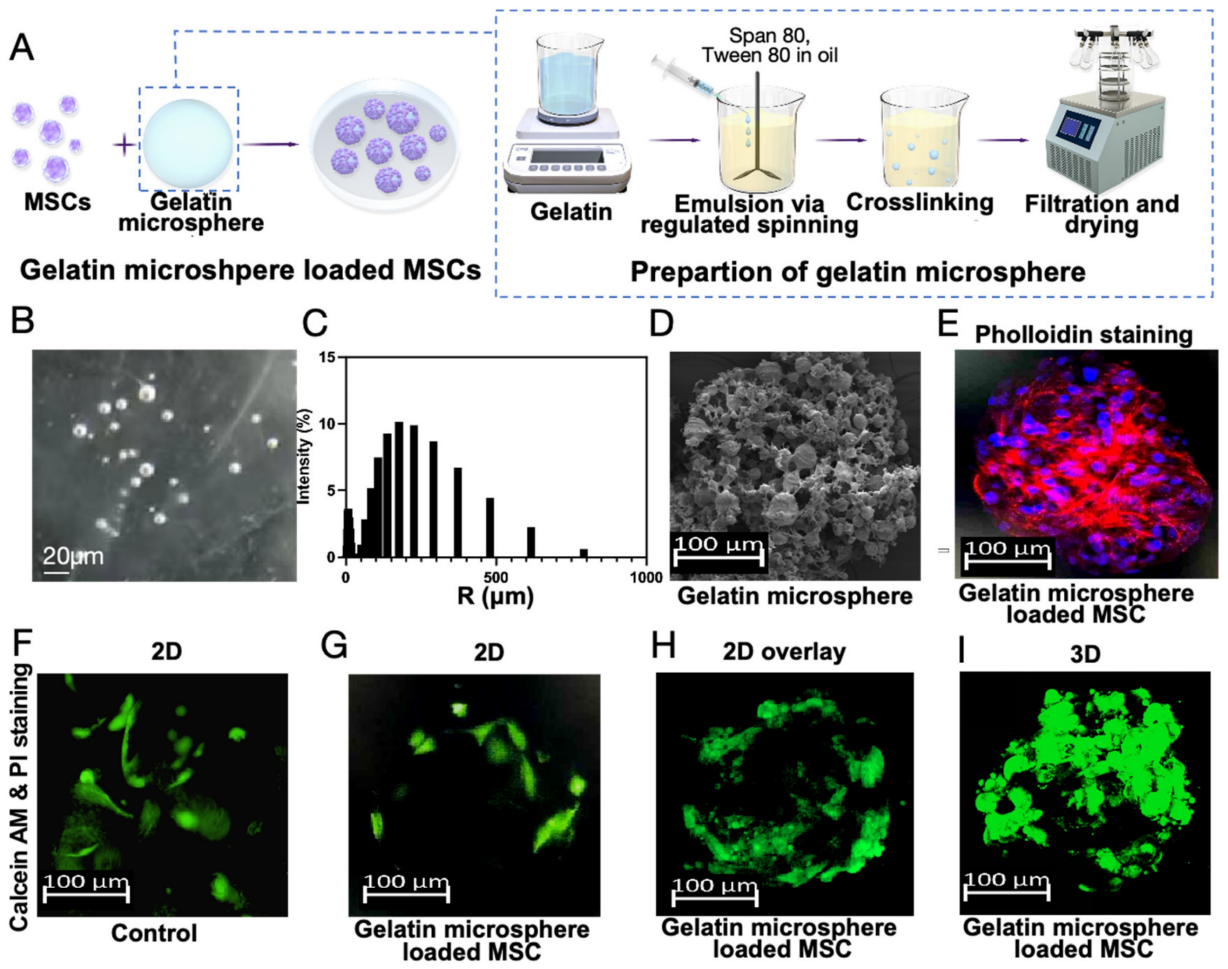
Preparation of Gelatin Microsphere and Three-Dimensional culture of MSCs. (A) Gelatin microsphere encapsulating MSCs. Schematic illustration of the gelatin composite preparation process. (B) Particle size detection of gelatin microsphere. (C) Statistical analysis of gelatin microsphere particle size distribution. (D) Scanning electron microscopy image of a gelatin microsphere. (E) MSCs were cultured with gelatin microsphere for 24h. Phalloidin staining of gelatin microsphere loaded with MSCs, highlighting the cytoskeleton. (F) Calcein AM and Propidium Iodide (PI) staining of microsphere loaded with MSCs, serving as the control group. (G) Two-dimensional fluorescence microscopy image of calcein AM and PI-stained microsphere loaded with MSCs. (H) Two-dimensional fluorescence overlay microscopy image of calcein AM and PI-stained microsphere loaded with MSCs. (I) Three-dimensional fluorescence microscopy image of calcein AM and PI-stained microsphere loaded with MSCs, demonstrating the three-dimensional distribution of MSCs within the microsphere.

Based on the above findings, this study aims to treat osteoarthritis by co-culturing gelatin microsphere with MSCs to regulate *Ext1* gene expression and modulate the immune response within the joint cavity. RGD sequence-modified polymers were mixed with gelling agents using a chemical cross-linking method to prepare three-dimensional gelatin microsphere, which facilitated cell adhesion due to the RGD sequence (Figure 3A). The diameter of microsphere is basically around 200 micrometers (Figure 3B-C). The gelatin microsphere, observed visually and under scanning electron microscopy (SEM), had a snowflake-like structure that effectively adhered to MSCs (Figure 3D). This structure could protect MSCs from internal environmental factors. The adhesion status of MSCs to the gelatin was observed using immunofluorescence staining. The gelatin microsphere helped maintain a three-dimensional culture state for the cells, which was closer to the *in vivo* cell growth environment (Figure 3E-I).

This study utilizes the combination of gelatin microsphere and MSCs to effectively deliver MSCs to the damaged areas of osteoarthritis using gelatin microsphere as a carrier. This acts like a protective shell for the MSCs, preventing them from being influenced by the internal environment of the joint cavity, such as the fluid shear stress that can damage cells. Additionally, the gelatin microsphere provides a three-dimensional culture environment for the cells, which, *in vitro*, has been shown to promote cell adhesion without affecting cell proliferation and growth. Gelatin microsphere are a unique carrier known for their biocompatibility, softness, and high water content among various biomaterials. They have been developed for different biomedical applications, such as drug delivery and tissue engineering^24^. Previous studies have used gelatin to treat osteoarthritis, where an increase in reactive oxygen species (ROS) in osteoarthritis led to the use of injectable calcium boride nanosheets (CBN) loaded hydrogel platform (CBN@GelDA hydrogel) as a high-payload and sustainable H_2_ precursor for OA treatment^25^. Recently, a one-component multifunctional polycitrate-based (PCCGA) hydrogel has been reported for the alleviation and cartilage protection of OA through its anti-inflammatory effects^26^. A simple, rapid, and efficient method has been used to form a self-assembled coating of TA-Sr^2+^ complexes on scaffolds, where the phenolic hydroxyl groups on the coating provide the meniscus scaffolds with excellent anti-inflammatory and reactive oxygen species scavenging abilities, thus treating osteoarthritis^27^. Additionally, MSCs have been used to treat osteoarthritis, where the exosomes secreted by MSCs act as intercellular messengers, carrying proteins, lipids, mRNA, miRNA, and other bioactive factors to treat OA in various ways^28^. In a Phase I/IIa clinical trial, patients with advanced Kellgren-Lawrence knee osteoarthritis received a single intra-articular injection of bone marrow-derived mesenchymal stem cells (BM-MSCs) to assess safety and efficacy. The Knee Injury and Osteoarthritis Outcome Score (KOOS) and Western Ontario and McMaster Universities Osteoarthritis Index (WOMAC) showed significant overall improvements in symptoms, quality of life, and WOMAC stiffness^29^. A high-yield exosome-like MSC-derived nanovesicles (MSC-NVs) with enhanced regenerative and anti-inflammatory capabilities have been developed. These not only induced M2 macrophage polarization but also promoted the differentiation, proliferation, and migration of chondrocytes and human bone marrow MSCs. In addition, a gelatin methacryloyl (GelMA)-NVs hydrogel loaded with MSC-NVs was prepared, which had a sustained release effect of MSC-NVs. GelMA-NVs is expected to treat osteoarthritis by regulating chondrogenesis and macrophage polarization^30^.

### Three-dimensional gelatin microsphere culture of MSCs promotes *Ext1* gene expression and immune response

**Figure 4:**
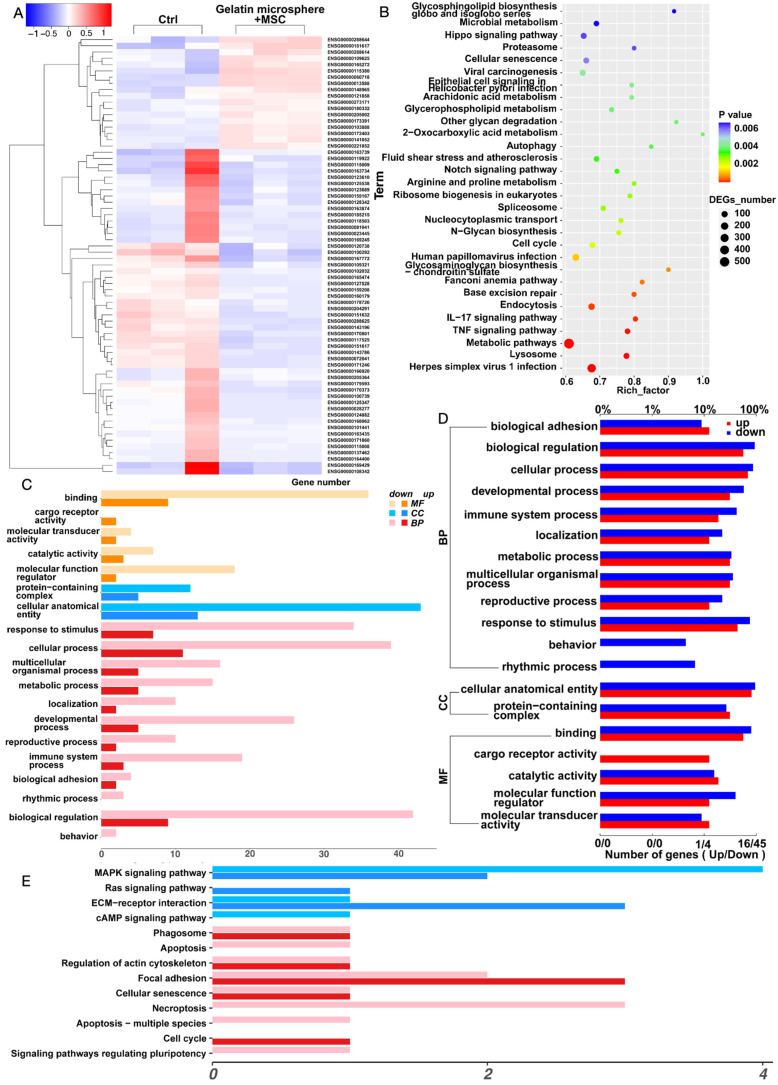
Enhanced Ext1 Gene Expression in MSCs via Three-Dimensional Culture with Gelatin Microsphere. (A) The control group consists of MSCs cultured in traditional two-dimensional cell culture dishes, while the experimental group involves MSCs loaded onto gelatin microsphere for three-dimensional cell culture. After 48 h of culturing, RNA was extracted from both groups for transcriptomic analysis. Heatmap illustrating the transcriptomic analysis of MSCs cultured in three dimensions with gelatin microsphere. (B) KEGG (Kyoto Encyclopedia of Genes and Genomes) enrichment plot of the transcriptomic data. (C) Plot of differentially expressed genes identified in the transcriptomic analysis. (D) GO (Gene Ontology) enrichment plot derived from the transcriptomic data. (E) KEGG pathway maps highlighting the biological pathways affected in the transcriptome of MSCs cultured with gelatin microsphere. BP: Biological Process. MF: Molecular Function. CC: Cellular Component.

We found that co-culturing MSCs with gelatin microsphere is beneficial for their three-dimensional growth. We further performed transcriptomic sequencing on the MSCs cultured in three dimensions with gelatin microsphere. We screened and analyzed genes that were upregulated more than 1.5-fold and downregulated more than 0.66-fold. First, we conducted a heatmap analysis of genes with differential expression (Figure 4A). KEGG (Kyoto Encyclopedia of Genes and Genomes) analysis suggested an increase in the expression of extracellular matrix (ECM) components, with the protein encoded by *Ext1* gene being expressed in the ECM pathway (Figure 4B). Functional analysis suggested immune system changed (Figure 4C). GO (Gene Ontology) analysis indicated an increase in cytokine-cytokine interactions, as well as enhanced expression of immune system processes and response to stimuli (Figure 4D). Pathway analysis showed ECM was altered, which was related with cartilage extracellular matrix (Figure 4E). These results suggest that three-dimensional cultured MSCs with gelatin microsphere promotes the expression of *Ext1* gene and simultaneously regulates the immune response.

Previous intra-articular injection of MSCs has been shown to be effective in treating OA and cartilage regeneration, but they may not be sufficient to completely repair articular cartilage defects. As shown in our results, future directions in improving MSCs treatments for osteoarthritis include genetic modification, complex products derived from MSCs extracellular vesicles, cell encapsulation in gelatin, and bioprinting 3D tissue engineering^31^. *In vivo* studies, a 20% PRP (platelet-rich plasma)-GelMA composite material was used for osteochondral reconstruction, and more cartilage and subchondral bone regeneration was observed than when using pure GelMA hydrogels. 3D printed PRP-GelMA composite materials can promote osteochondral repair through M2-polarized immune regulation. The PRP-GelMA hydrogel promotes the migration and osteogenic/chondrogenic differentiation of bone marrow mesenchymal stem cells and is involved in the immune regulation of macrophages and M1-M2 transformation^32^.

### IFN-γ induced MSCs cultured with gelatin microsphere to exhibit immunomodulatory capabilities

**Figure 5:**
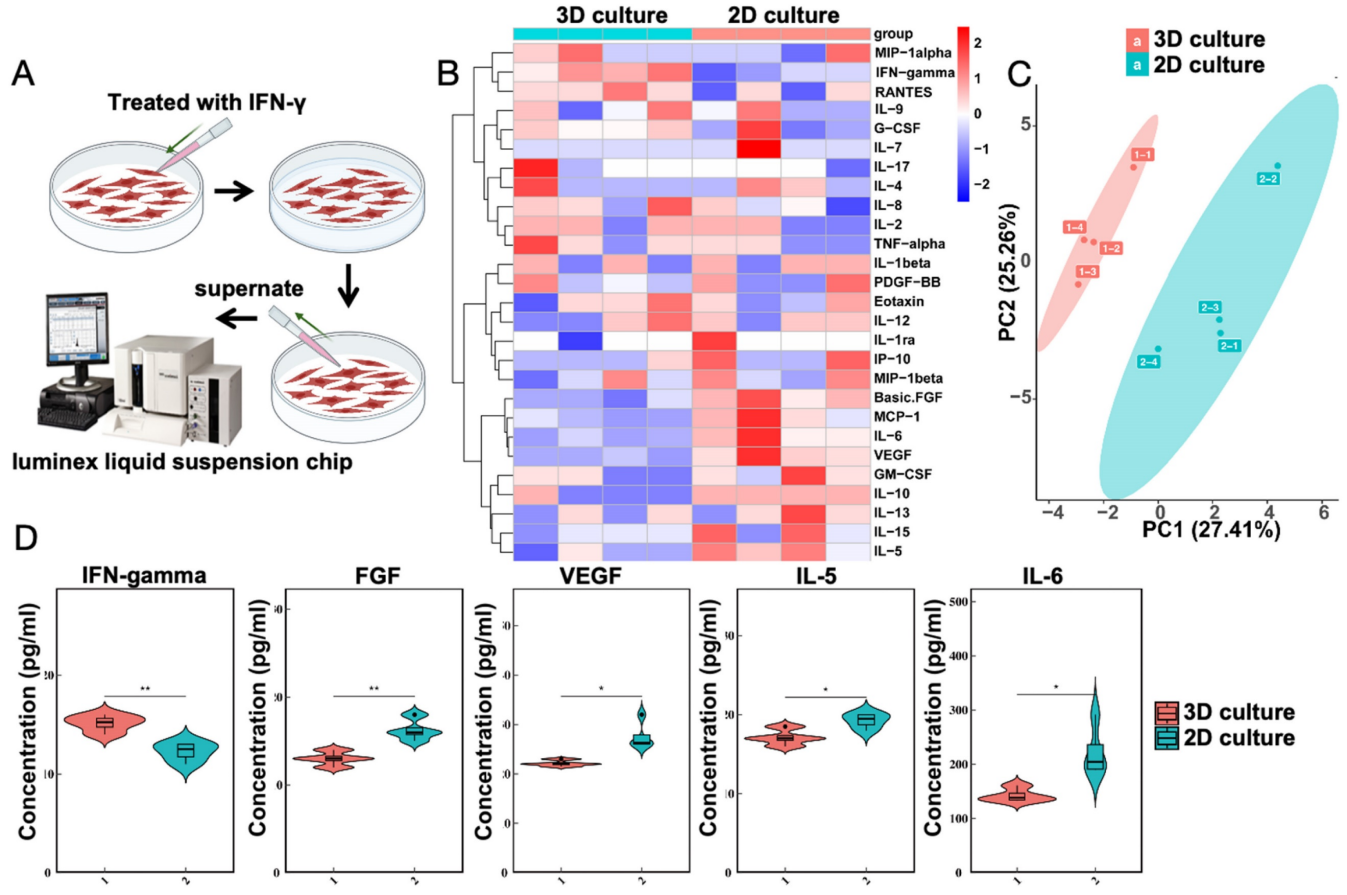
IFN-γ Enhances Immunomodulatory Properties of MSCs was cultured with Microsphere. (A) The control group consists of MSCs, while the experimental group involves cultured gelatin microsphere with MSCs. After 24 h of culturing, IFN-γ was added to both groups. Following a 24 h intervention, the supernatant was collected from the cells and subjected to Luminex liquid suspension chip immune cytokine analysis. Schematic representation of IFN-γ treatment on MSCs. (B) Heatmap depicting the analysis of cytokine expression. (C) Principal Component Analysis (PCA) results of the cytokine expression analysis. (D) Changes in inflammatory factor levels as determined by cytokine analysis.

The transcriptomic results suggest an increase in cytokine-cytokine interactions. To test the immunomodulatory capabilities of MSCs cultured with gelatin microsphere, the gelatin microsphere adhered MSCs were exposed to the pro-inflammatory cytokine IFN-γ. Both groups consist of MSCs cultured with gelatin microspheres. The control group is not treated with IFN-γ, while the experimental group is treated with IFN intervention (Figure 5A). Twenty-five related inflammatory factors were detected (Figure 5B), and it was found that after IFN-γ stimulation, the expression of five inflammatory factors changed (Figure 5C), including IFN-γ, basic FGF, VEGF, IL-5, and IL-6 (Figure 5D). The results of the liquid phase suspension chip indicated that the gelatin microsphere adhered MSCs enhanced their immunomodulatory efficacy.

Osteoarthritis often involves immune dysregulation, such as factors like interleukins, TGF, and helper T cells^33^. Immunomodulatory nanoparticles targeting osteoarthritis can effectively alleviate the symptoms of osteoarthritis^34^. Intra-articular injection of MSCs into patients with advanced osteoarthritis can alleviate symptoms, after MSCs injection, the levels of pro-inflammatory monocytes/macrophages and interleukin-12 in the synovial fluid decrease^29^. Recent studies have found that *Ext1* gene is associated with immunity; a mutant mouse lacking *Ext1* enzyme, which is essential for the synthesis of heparan sulfate, was produced in a Tek-dependent and induced manner. The mutant mice exhibited severe lymphocyte homing impairment and contact hypersensitivity impairment, demonstrating the key role of endothelial cell heparan sulfate in immune surveillance and immune response generation^35^. *Ext1* mutations have been detected in patients with membranous nephropathy, and autoimmune diseases are common in these patients^36^. *Ext1* on the glomerular basement membrane has been reported as a new putative antigen for membranous nephropathy with autoimmune disease, and the serum complement levels and IL-4/IFN-γ ratio are increased in *Ext1*-positive membranous lupus nephritis^37^. Studies have found that heparan sulfate is most abundantly expressed in thymic fibroblasts, and eliminating most heparan sulfate in the organ by genetically disrupting the synthesis of the necessary glycosyltransferase *Ext1* greatly reduces the size of fetal thymus organ culture and mouse thymus, reduces T cell production, disrupts the CCL19/CCL21 chemokine gradient, and impairs dendritic cell migration in thymus sections^38^. Lymphatic endothelial heparan sulfate is essential for the formation of a functional CCL21 gradient^39^.

### Rat model with knee osteoarthritis confirmed that gelatin microsphere cultured with MSCs helps alleviate osteoarthritis *in vivo*

**Figure 6:**
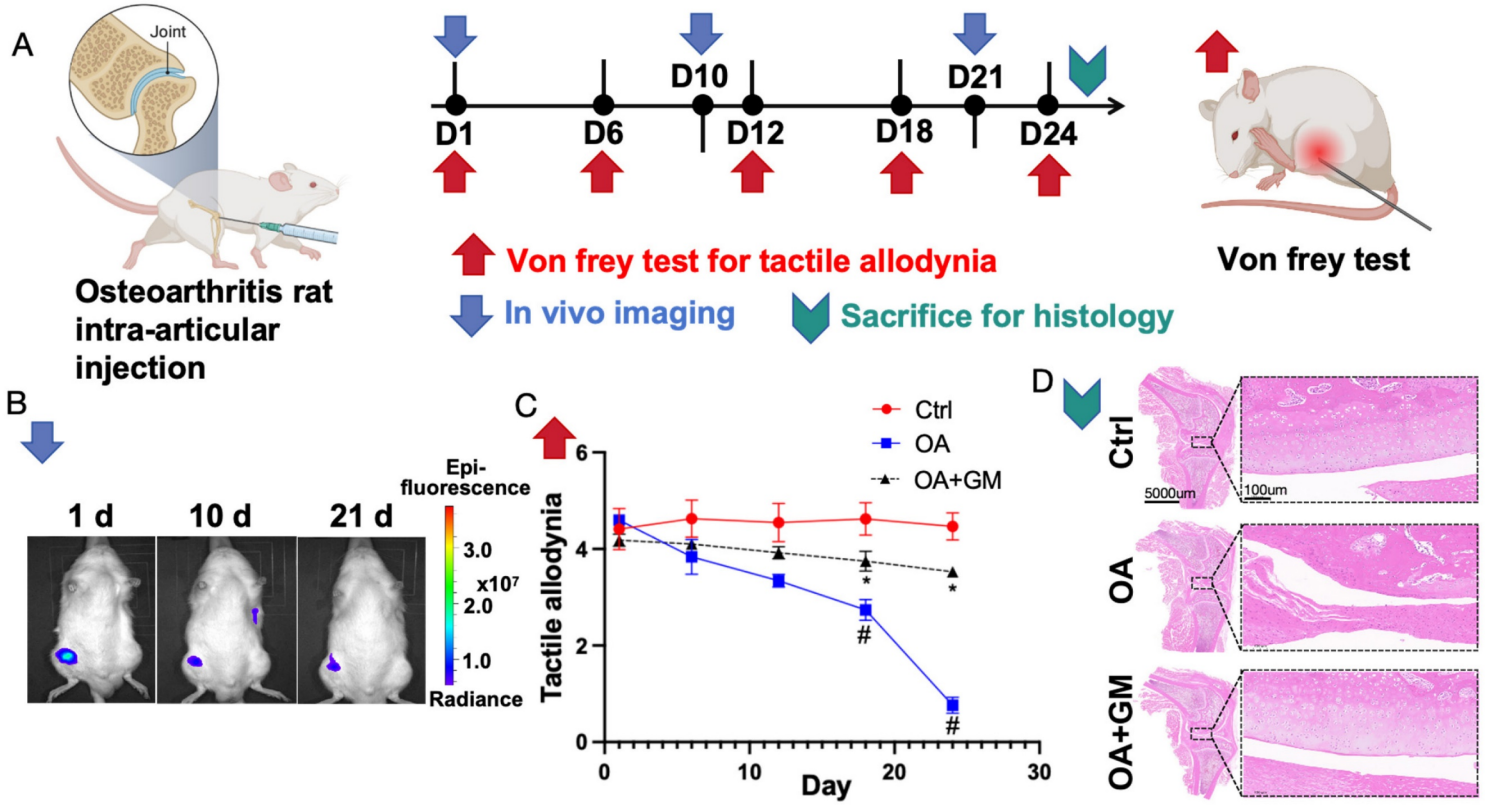
Cultured MSCs and gelatin microsphere (GM) is beneficial to alleviate Osteoarthritis (OA) *in vivo*. (A) Timeline of *in vivo* experiments. Osteoarthritis rat models were made by anterior cruciate ligament transected. Day 1 was defined by 8 weeks after rat OA model established. Gelatin microsphere loaded with MSCs were intra-articular injected at Day1 (1d). Von Frey test measures knee joint pain sensitivity by applying filaments of different sizes to gauge mechanical pain thresholds. (B) Following intra-articular injection of gelatin microsphere loaded with MSCs. *In vivo* imaging was measured by 1^st^ day (1d), 10^th^ day (10d) and 21^th^ day (21d). Living cells were labeled by DiR Iodide. The labeled cells appear to be a darker color in the color scheme. (C) Von Frey test of rat knee joint was measured by 1^st^ day (1d), 6^th^ day (6d), 12^th^ day (12d), 18^th^ day (18d) and 24^th^ day (24d). Von Frey test was used to assess joint pain by applying varying forces to the skin around the joint to determine pain threshold sensitivity. Lower tactile allodynia means more painful. (D) Rats were sacrificed by 24^th^ day. Representative H&E staining image of knee joints for Control, OA and OA+GM group.

To evaluate the biosafety of the gelatin upon its entry into the body, an experiment was conducted where the gelatin microsphere was cultured with MSCs was injected into the knee cavity of wild-type rats (Figure 6A). *In vivo* imaging detection demonstrated that the cells could survive for more than 21 days within the joint cavity without migrating to other tissues outside the joint cavity (Figure 6B). Additionally, no significant abnormalities were observed in the peripheral blood of the rats. Von Frey pain assessment reveals that enhancing the injection of gelatin microsphere and MSCs into the osteoarthritic joint cavity can alleviate knee joint pain (Figure 6C). Histological examination revealed that the gelatin microsphere did not damage the normal cartilage tissue upon entering the joint cavity (Figure 6D). These findings confirm the safety of *in vivo* treatment with gelatin microsphere. This will provide new strategies for tissue injury repair^40^.

Gelatin microsphere show significant potential in treating and preventing OA, warranting further clinical research in this innovative tissue engineering approach^41^. To validate the therapeutic effect of the gelatin microsphere cultured with MSCs on osteoarthritis, we constructed a rat knee osteoarthritis model by damaging the anterior cruciate ligament. Von Frey testing indicated that injection of the gelatin microsphere cultured with MSCs could alleviate the pain tactile allodynia associated with osteoarthritis. Histological examinations revealed that the gelatin microsphere cultured with MSCs could mitigate the process of cartilage damage in osteoarthritis and protect the structural morphology of the knee joint cartilage. The *in vivo* experimental results suggest that the gelatin microsphere cultured with MSCs can effectively treat knee joint osteoarthritis.

## Conclusions

We found that the expression of *Ext1* gene in the cartilage weight-bearing area of patients with osteoarthritis was weakened and used the gelatin microsphere loaded MSCs treatment can promote MSCs overexpression of *Ext1* gene, alleviate OA osteoarthritis, and regulate intra-articular immune response *in vivo*.

First, during the surgical process of patients with advanced osteoarthritis, cartilage tissues were obtained from both weight-bearing and non weight-bearing areas. Transcriptomic analysis showed that the expression of *Ext1* gene in the weight-bearing area was downregulated, while the expression of immune regulatory factors was upregulated.

Second, constructing a gelatin microsphere based MSCs system, *in vitro* experiments have confirmed that gelatin microsphere has good biocompatibility, which is beneficial for the three-dimensional culture of MSC cells and can simulate the three-dimensional growth environment of cells *in vivo*. The porous structure of gelatin microsphere is conducive to cell metabolism and secretion. Transcriptomic analysis of cells suggests that the three-dimensional culture state can promote the overexpression of *Ext1* in MSCs, and also regulate immune response.

Third, *in vivo* experiments were conducted to construct an OA rat model, and gelatin microsphere was loaded with MSCs and injected into the joint cavity, which has biological safety and lasts for 30 days in the joint cavity.

In summary, the decreased expression of the Ext1 gene in cartilage of patients with osteoarthritis in a clinical issue. To solve this problem, gelatin microsphere loaded MSCs were constructed through biomaterial engineering. This system mimics the three-dimensional growth pattern of cells *in vivo*, promotes overexpression of *Ext1* gene, and regulates the immune response in the joint cavity, achieving the effect of treating knee osteoarthritis.

This study provides a new direction for the future clinical treatment of osteoarthritis by constructing a gelatin microsphere MSCs system. The gelatin microsphere MSCs system for osteoarthritis treatment shows promise for personalized, non-surgical therapy, reducing reliance on medications, improving joint health, and offering a cost-effective solution.

## Supplementary Material

Supplementary figures.
