# Supplementary material for: Free glutaraldehyde gelatin microsphere loaded mesenchymal stem cells alleviate osteoarthritis by promoting Ext1 expression
Source: Theranostics. 2025 Mar 10;15(9):4135–46. doi: 10.7150/thno.109468 (PMC11980673; doi:10.7150/thno.109468)
Supplement: Supplementary file 1 — Supplementary figures. [file thnov15p4135s1.pdf]

In order to further substantiate the findings from the transcriptomic sequencing data, we prospectively enrolled an additional seven patients with osteoarthritis who were undergoing total knee arthroplasty at our institution. During the surgical procedure, discarded cartilaginous tissue from the femoral condyles was harvested and categorized into two distinct regions: the remote zone and the lesion zone. The cartilage from the remote zone was designated as the control cohort, whereas the cartilage from the lesion zone was allocated to the experimental cohort. RNA was isolated from the cartilage samples of both cohorts, followed by reverse transcription to cDNA. Subsequent polymerase chain reaction (PCR) analysis was conducted to quantify the expression levels of the Ext1 gene. The analysis revealed a significant downregulation of the ext1 gene expression in the lesion zone cartilage, thereby corroborating the results obtained from the transcriptomic analysis (Figure S1).

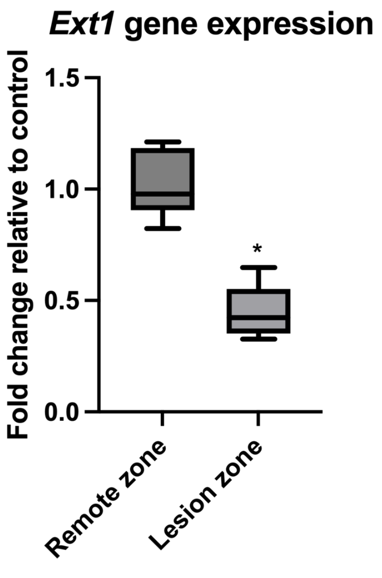

Figure S1 The RT-PCR results suggest that the expression of the Ext1 gene in the cartilage of the remote zone is downregulated compared to the lesion zone.
